# Antigen-Specific CD4^+^ T Cells Exhibit Distinct Kinetic and Phenotypic Patterns During Primary and Secondary Responses to Infection

**DOI:** 10.3389/fimmu.2020.02125

**Published:** 2020-09-02

**Authors:** Deepali Malhotra, Kristina S. Burrack, Marc K. Jenkins, Anne E. Frosch

**Affiliations:** ^1^Department of Microbiology and Immunology, Center for Immunology, University of Minnesota, Minneapolis, MN, United States; ^2^Hennepin Healthcare Research Institute, Minneapolis, MN, United States; ^3^Department of Medicine, University of Minnesota, Minneapolis, MN, United States

**Keywords:** CD4 T cell, *Listeria monocytogenes*, secondary response, CD49a, CD69, Ly6C

## Abstract

Although CD4^+^ T cell memory is a critical component of adaptive immunity, antigen-specific CD4^+^ T cell recall responses to secondary infection have been inadequately studied. Here we examine the kinetics of the secondary response in an important immunological model, infection with attenuated *Listeria monocytogenes* (Lm). We identify CD4^+^ T cell subsets that preferentially expand during a secondary response and highlight the importance of prime-boost strategies in expanding and maintaining antigen-specific, tissue-resident memory CD4^+^ T cells. Following intravenous infection with an attenuated strain of Lm, we found that total antigen-specific CD4^+^ T cells responded more robustly in secondary compared with primary infection, reaching near-peak levels in secondary lymphoid organs (SLOs) and the liver by three days post-infection. During the secondary response, CD4^+^ T cells also contracted more quickly. Primary Lm infection generated two main classes of effector cells: Th1 cells that assist macrophages and T follicular helper (Tfh) cells that aid B cells in antibody production. We found that during the secondary response, a population of Ly6C^+^ Tfh cells emerged in SLOs and was the basis for the skewing of this response to a Tfh phenotype. Deletion of T-bet in T cells precluded development of Ly6C^+^ Tfh cells, but did not alter anti-Lm antibody responses. Moreover, during recall responses, CD49a^+^ Th1 cells preferentially expanded and accumulated in the liver, achieving a new set point. Parabiosis experiments indicated that, in contrast to Tfh cells and most splenic Th1 cells, the majority of CD49a^+^ Th1 cells in the liver were tissue resident. Overall, these data demonstrate a robust secondary CD4^+^ T cell response that differs in kinetics and composition from the primary response and provide insight into targets to enhance both peripheral and tissue-resident CD4^+^ T cell responses.

## Introduction

The ability of CD4^+^ T cells to generate memory is a critical feature of their biology and is thought to allow the immune system to respond more rapidly and effectively to a previously encountered infection. With infections that become chronic or recurrent, there may also be utility in blunting an inflammatory immune response that may be damaging to the host. An acute CD4^+^ T cell response may be comprised of various effector populations including T helper 1 (Th1), Th2, Th17, regulatory T (Treg), and T follicular helper (Tfh) cells. The distribution of a naïve helper T cell population into these subsets is guided by innate signaling as well as TCR signaling during the primary infection. The degree to which these initial transcriptional fates in a primary infection dictate the phenotype of a memory cell’s progeny during a secondary response has been intensely investigated. Adoptive transfer studies have transformed the study of these antigen specific T cell populations, providing insight into the fate of CD4^+^ T cell populations (1–3). Research utilizing these techniques suggests that memory CD4^+^ T cell populations retain qualitative aspects of the primary immune response (transcriptional profile and epigenetic modification) but maintain plasticity in a population of central memory T cells that express CCR7 (4, 5). While there has been tremendous interest in the lineage of these distinct CD4^+^ T cell subsets in memory, there has been minimal characterization of an actual intact memory response relative to the primary response.

*Listeria monocytogenes* (Lm) is a gram-positive bacillus that causes a primary gastrointestinal infection in humans that can disseminate via the blood stream to distant sites. With the tools available for tracking antigen-specific T cell populations in this infection, Lm has been a central model for studying CD4^+^ T cell immunity in murine models. Primary infections both in mice and humans are characterized by a strong inflammatory response (6). Accordingly, the primary CD4^+^ T cell response in a Lm infection is comprised primarily of Th1 and Tfh cells (7). We sought to investigate the antigen (Ag)-specific CD4^+^ T cell response to Lm infection following primary and secondary (recall) infection to understand how CD4^+^ T cell memory evolves in the course of these responses. We found that Ag-specific CD4^+^ T cells expanded more quickly in the secondary lymphoid organs (SLOs) and liver during the secondary response, reaching a new set point in the liver, a site of intravenous Lm infection. With increasing evidence of the importance of residency in CD4^+^ T cell memory, we stained for CD49a, which, similar to CD69 (8), has been used to define tissue residence in macrophages, NK cells, CD4^+^ T cells, and CD8^+^ T cells (9–14). We identified a subset of CD49a^+^CD69^+^ Th1 cells that preferentially expanded during secondary infection and maintained residence in the liver, in constrast to Tfh cells and most splenic Th1 cells, which efficiently recirculated. Moreover, we describe a subset of Tfh cells that specifically emerge in SLOs during secondary Lm infection and exhibit elevated Ly6C expression, which was dependent on expression of T-bet. Overall, these data demonstrate the accelerated kinetics and altered differentiation associated with a secondary response to Lm and emphasize the importance of boosting for expanding tissue-resident memory CD4^+^ T cells.

## Materials and Methods

### Mice

C57BL/6 mice were obtained from the National Cancer Institute. T-bet^F/F^ and Lck-Cre mice were purchased from Jackson Laboratories and bred in-house. Animals were housed in specific pathogen-free conditions at the University of Minnesota. The Institutional Animal Care and Use Committee at the University of Minnesota approved all experimental procedures.

### Infections

All mice were injected intravenously with 5 × 10^7^ colony forming units (CFU) of an *actA-*deficient Lm bacteria engineered to secrete a fusion protein containing an immunogenic peptide called 2W (Lm-2W) (15). Where relevant, mice were re-infected with the same dose and strain of Lm-2W after at least 45 days had passed since the primary infection. In order to standardize dosing, a single large batch of Lm-2W was grown, aliquoted, and frozen in small batches for infection. Aliquots were thawed, diluted, and rapidly administered intravenously to mice in order to ensure equal dosing with Lm-2W. All aliquots were prepared for single use (one thaw).

### Flow Cytometry (Harvests, Tissue Processing, Flow)

On days 0, 3, 5, 7, 14, 21, and 32 post-infection, mice were euthanized and SLOs (spleen and lymph nodes), liver, and blood were harvested for analysis of Ag-specific CD4^+^ T cells. Prior to euthanasia, anti-CD45.2 antibody (PE-Cy7, clone 104, BD Biosciences) was injected intravenously in order to distinguish vascular-associated cells from tissue-resident cells specifically in the liver. Secondary lymphoid organ and liver tissues were homogenized to single-cell suspensions. Blood samples underwent red blood cell lysis. Following enrichment using fluorophore-conjugated tetramers for 2W and/or listeriolysin O (LLO) peptide (2W:I-A^b^, LLOp:I-A^b^) (1), cells were stained for the following surface markers: CD90.2 (AF700, clone 30-H12, BioLegend), CD4 (BUV395, clone GK1.5, BD Biosciences), CXCR5 (BV650, clone L138D7, BioLegend), CD49a (BV711, clone Ha31/8, BD Biosciences), Ly6C (BV785, clone HK1.4, BioLegend), CD11b (APC-eF780, clone M1/70, eBioscience), CD11c (APC-eF780, clone N418, eBioscience), B220 (APC-eF780, clone RA3-6B2, eBioscience), F4/80 (APC-eF780, clone BM8, eBioscience), CD69 (PE, clone H1.2F3, BioLegend), CD44 (V500, clone IM7, BD Biosciences), and a live/dead marker (Ghost Dye 780, TONBO biosciences). Following fixation and permeabilization (Foxp3 Fix/Perm Kit, eBioscience/ThermoFisher), cells were stained for intracellular markers: T-bet (BV605, clone 4B10, BioLegend) and Foxp3 (PerCP-Cy5.5, clone FJK-16s, eBioscience). Foxp3 staining was performed to exclude a small population (typically <5%) of Ag-specific Treg cells that is found after Lm infection. Cells were acquired on a LSRFortessa Flow Cytometer (BD Biosciences) and analyzed using FlowJo (version 9). Ag-specific CD4^+^ T cells were identified as follows, as previously described (16): gate for lymphocytes; then a gate for single cells; then a gate for cells positive for CD90.2 and negative for CD11b, CD11c, B220, F4/80, and the dead cell marker; then a gate for cells positive for CD4; then a gate for CD44^hi^ tetramer-binding cells. From here, Treg cells were excluded on the basis of Foxp3 staining. For analysis of the liver, a gate was applied prior to analysis of CD4 expression to exclude vasculature-associated T cells on the basis of CD45.2 staining in intravenously injected mice.

### Parabiotic Surgeries

Parabiotic surgery was performed as described (17). Briefly, mice with discordant congenic markers, CD45.1 and CD45.2 were anesthetized with ketamine and xylazine. Matching incisions were made in shaved and disinfected skin extending from the olecranon to the knee joint paired mice. Continuous staples were used to appose the skin. Parabionts were then allowed to rest for 18–19 days before experiments. Peripheral blood was used to confirm equilibration before tissue analysis.

### Serological Analysis

All animals were sacrificed by CO_2_ inhalation, and blood was immediately collected by cardiac puncture. Serum samples were separated by centrifugation in tubes containing EDTA and stored at −80°C until analyzed. Individual serum samples were analyzed separately by enzyme-linked immunosorbent assays (ELISA). For all ELISAs, 96-well plates were coated with 1 μg of LLO (Abcam) or 1 × 10^7^ CFU of heat-killed Lm per well and incubated at 37°C for 1 h. All subsequent steps were conducted at room temperature. After blocking non-specific sites with 10% fetal calf serum, sera from experimental animals were added at a dilution of 1:50 for analysis of Lm-specific IgG or 1:10 for analysis of LLO-specific IgG and Lm-specific IgM, IgG2a and IgG2c. Horseradish peroxidase-conjugated rabbit anti-mouse IgG, IgM, IgG2a, and IgG2c (Invitrogen) was used for determination of the total concentration of IgG in serum. The optical density at 405 nm was determined with an ELISA reader (Bio-Tek Instruments) after addition of 100 μl of a 1× solution of KPL ABTS substrate solution (SeraCare).

### Statistical Analysis

The D’Agostino and Pearson omnibus normality test was used to evaluate if data followed a normal distribution. For data with a normal distribution, analysis was conducted with parametric tests (two group analyses – unpaired two-tailed Student’s *t* test; greater than two groups – one-way ANOVA with Tukey’s multiple comparison tests). For data with a non-normal distribution, non-parametric tests were used (two group analyses – Mann–Whitney *U* test, greater than two groups – Kruskal–Wallis with Dunn’s multiple comparison test) unless otherwise stated. *P* < 0.05 was considered significant. Statistical analyses were done using GraphPad Prism (GraphPad Software). No sample exclusion criteria were applied to datasets. Mice were not randomized for group allocation, and investigators were not blinded to intervention group.

## Results

### Ag-Specific CD4^+^ T Cells Expand and Contract More Rapidly Following Secondary Infection and Exhibit a Smaller Proliferative Capacity

**FIGURE 1:**
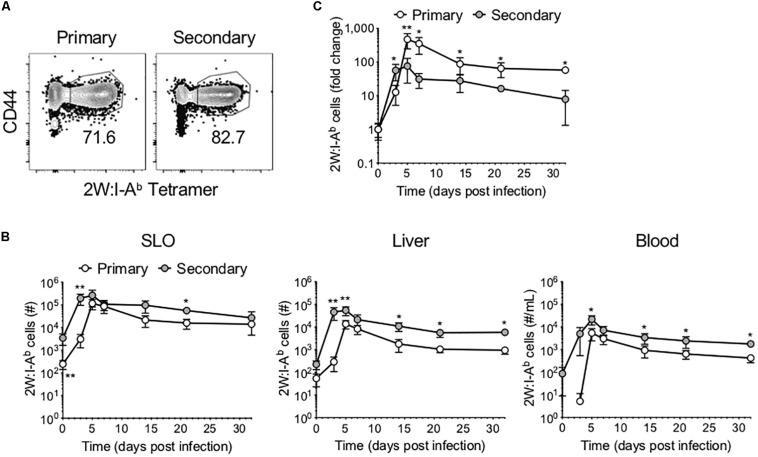
Ag-specific CD4^+^ T cells expand and contract more rapidly following secondary infection. Following primary or secondary Lm infection, the number of 2W:I-A^b^-specific CD4^+^ T cells within secondary lymphoid organs (SLOs), liver and blood was assessed at 0, 3, 5, 7, 14, 21, and 32 dpi. **(A)** Representative flow cytometry plots of 2W:I-A^b^-specific CD4^+^ T cells in SLOs at 5 dpi, after excluding doublets, dead cells, B cells, macrophages, dendritic cells, and CD8^+^ T cells as described in the materials and methods. **(B)** Number of 2W:I-A^b^-specific CD4^+^ T cells in SLO, liver and blood. **(C)** Fold change of 2W:I-A^b^-specific CD4^+^ T cell expansion during primary versus secondary Lm infection. Data are compiled from two to three independent experiments per time point with *n* = 1–4 mice per experiment. *P < 0.05, **P < 0.01 as determined by Mann Whitney *U* test. Bars represent mean ± SD.

We sought to compare the phenotype and function of Ag-specific CD4^+^ T cells following primary versus secondary Lm infection. To this end, mice were infected with Lm-2W, an attenuated Lm strain engineered to secrete a fusion protein containing the immunogenic 2W peptide (which is presented in the context of I-A^b^) and containing a mutation in the *actA* gene that inhibits intracellular movement of the bacteria (18). A subset of mice was re-infected with the same dose of Lm-2W at a memory time point (>45 days post-infection, dpi) (Supplementary Figure S1). Mice were euthanized on days 3, 5, 7, 14, 21, and 32 post-infection, and the 2W-specific CD4^+^ T cell response was assessed in the SLOs, liver, and blood using flow cytometry following enrichment for Ag-specific CD4^+^ T cells using 2W:I-A^b^ tetramer and magnetic bead technology (19) (Figure 1A). We found that the 2W-specific CD4^+^ T cells expanded more rapidly following secondary infection, reaching near peak levels as early as 3 dpi in the SLOs, blood, and liver (Figure 1B). Further, the number of 2W-specific CD4^+^ T cells reached a new set point in the liver following secondary infection, highlighting the ability of prime-boost strategies to bolster Ag-specific CD4^+^ T cell numbers for extended periods in non-lymphoid tissues. Although 2W-specific CD4^+^ T cells expanded more rapidly during the secondary response, when cell counts were normalized to the population size at the time of infection (“0” dpi), the Ag-specific CD4^+^ T cell population proliferated to a larger degree during the primary infection than secondary infection (Figure 1C). At 3 dpi the number of 2W-specific CD4^+^ T cells had increased by 50-fold in the secondary response as compared to 10-fold in the primary response. But by 5 dpi, the number of 2W-specific CD4^+^ T cells had increased by 75-fold in the secondary response versus 472-fold in the primary. At 7 dpi, the number of 2W-specific CD4^+^ T cells in Lm-infected mice during the primary response was still 350-fold greater than in naïve mice, whereas the number of 2W-specific CD4^+^ T cells in the secondary response was only 31-fold greater than at time “0”. Thus, the Ag-specific CD4^+^ T cell response contracted more quickly during the secondary response than the primary; however, this may be expected due to more rapid clearance of Lm during secondary infection.

**FIGURE 2:**
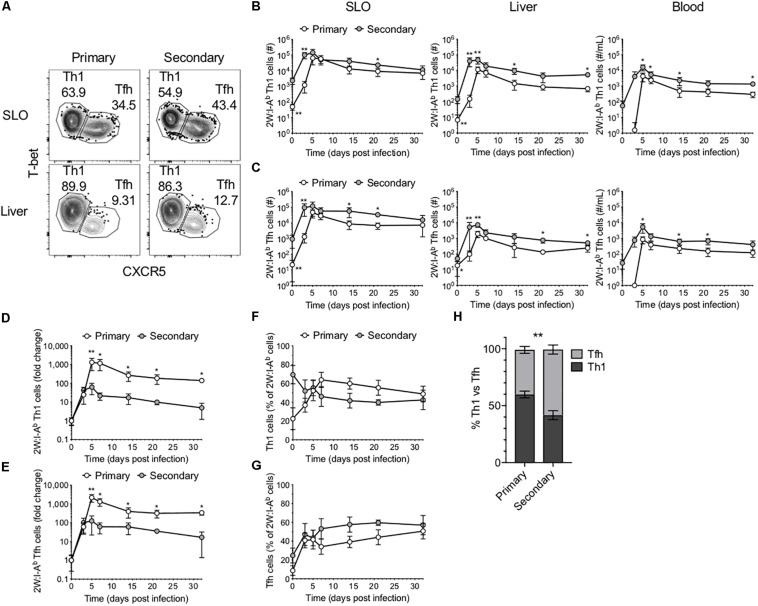
Comparison of Th1 and Tfh responses in primary and secondary Lm infection. **(A)** Phenotype of 2W:I-A^b^-specific CD4^+^ T cells in the SLOs and liver at 5 dpi following primary or secondary Lm infection after excluding Foxp3^+^ Tregs. Number of 2W:I-A^b^-specific T-bet^+^ Th1 cells **(B)** and CXCR5^+^ Tfh cells **(C)** in the SLOs, liver and blood. Fold change of 2W:I-A^b^-specific Th1 **(D)** or Tfh **(E)** cell expansion during primary versus secondary Lm infection. Frequency of 2W:I-A^b^-specific T-bet^+^ Th1 cells **(F)** or CXCR5^+^ Tfh cells **(G)** in SLOs. **(H)** Frequency of 2W:I-A^b^-specific Th1 versus Tfh cells in SLOs at 14 dpi. Data are compiled from two to three independent experiments per time point with *n* = 1–4 mice per experiment. *P < 0.05, **P < 0.01 as determined by two-way ANOVA followed by Sidak’s multiple comparison test **(H)** or Mann Whitney *U* test **(B–G)**. Bars represent mean ± SD.

As mentioned, CD4^+^ T cell responses to Lm have been well described as being primarily comprised of Th1 and Tfh cells. To investigate the phenotype of the 2W-specific CD4^+^ T cells, we assessed T-bet and CXCR5 expression as markers of Th1 and Tfh cells, respectively (3, 4, 7) (Figures 2A–C). Of note, there is evidence that CXCR5 may not reliably label Tfh cells in the setting of lymphocytic choriomeningitis virus (LCMV) infection (20). However, these markers were selected on the basis of previous work in the Lm-2W model that demonstrated that the generation of CXCR5^+^ cells is strictly dependent on Bcl6 expression and that CXCR5 reliably identifies two populations during acute infections: PD1^low^ Tfh cells and PD1^hi^ GC-Tfh cells (3, 4, 7). Further, at memory time points in the Lm-2W model, a single CXCR5^+^ Tfh population is identified that gives rise to CXCR5^+^ progeny during single cell recall experiments. In our primary versus recall infections, the total number of 2W-specific Th1 and Tfh cells expanded more quickly during the secondary response (Figures 2B,C). However, similar to the total 2W-specific CD4^+^ T cell response, the fold change of the 2W-specific Th1 and Tfh cells was greater by 5 dpi during the primary response (Figures 2D,E). These data indicate that following secondary Lm infection, the Ag-specific CD4^+^ T cell response expands and contracts more rapidly and overall undergoes less proliferation than during the primary response. Furthermore, while the ratio of Th1 to Tfh cells was similar in SLOs early after infection (3–5 dpi) during the primary and secondary responses, despite the likely rapid clearance of Lm antigen, the secondary 2W-specific CD4^+^ T cell response surprisingly (7) favored a Tfh phenotype between days 7 and 21 post-infection (Figures 2F–H). The ratio of Th1:Tfh cells was approximately 60:40 during the primary response but shifted to 40:60 during the secondary response (Figure 2H), indicating that the secondary CD4^+^ T cell response in SLOs was skewed to a Tfh-like phenotype during that time window. These data reflect the continual evolution of the Ag-specific CD4^+^ T cell response upon re-exposure to the bacterial pathogen Lm. The observed skewing of the secondary response in SLOs to a Tfh phenotype may be indicative of heterogeneity in this population of effectors and a concomitant outgrowth of a Tfh subset.

### Ly6C^+^ Tfh Cells Expand in SLOs During the Secondary Response and Require T-bet

**FIGURE 3:**
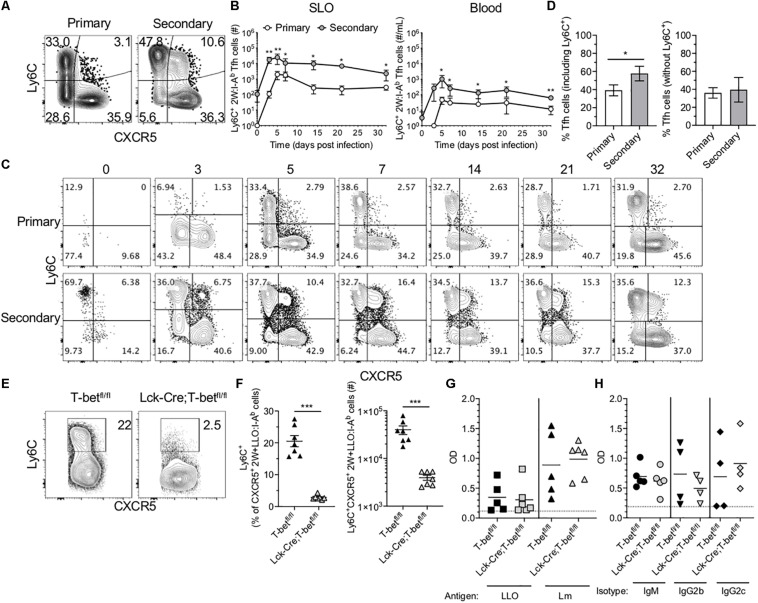
Ly6C^+^ Tfh cells expand during the secondary response and require T-bet. **(A)** Representative flow cytometry plots demonstrating CXCR5 and Ly6C expression on 2W:I-A^b^-specific CD4^+^ T cells, excluding Foxp3^+^ Tregs, in the SLOs at 5 dpi following primary or secondary Lm infection. **(B)** Number of Ly6C^+^ Tfh cells in SLO and blood. **(C)** Representative flow cytometry plots of CXCR5 and Ly6C expression on 2W:I-A^b^-specific cells, excluding Foxp3^+^ Tregs, in the SLOs at 0, 3, 5, 7, 14, 21, and 32 dpi following primary or secondary Lm infection. **(D)** Frequency of Tfh cells in the SLOs at 14 dpi including or excluding Ly6C^+^ cells. **(E)** Representative flow cytometry plots demonstrating Ly6C expression on 2W + LLO:I-A^b^-specific CXCR5^+^ Tfh cells in the SLOs following secondary Lm infection of T-bet^fl/fl^ or Lck-Cre;T-bet^fl/fl^ mice at 5 dpi. **(F)** Frequency and number of Ly6C^+^ Tfh cells as shown in panel **(E)**. **(G)** ELISA data assessing the presence of anti-LLO or anti-Lm IgG antibodies in the serum of T-bet^fl/fl^ or Lck-Cre;T-bet^fl/fl^ mice following secondary Lm infection at 10 dpi. **(H)** ELISA data assessing the presence of anti-Lm IgM, IgG2b or IgG2c antibodies as in panel **(G)**. Data are compiled from two to three independent experiments per time point with *n* = 1–4 mice per experiment **(A–D)** or from two independent experiments with *n* = 2–4 mice per group per experiment **(E–H)**. *P < 0.05, **P < 0.01, ***P < 0.001, ****P < 0.0001 as determined by unpaired Student’s *t*-test **(D,F)** or Mann Whitney *U* test **(B)**. Bars represent mean ± SEM **(D,F)** or mean ± SD **(B)**.

In light of the Tfh-skewing of secondary 2W-specific CD4^+^ T cell responses in SLOs, the potential for heterogeneity within this population was investigated. Ly6C expression has been used to distinguish effector and memory subsets within CD4^+^ T cells (21). Further, existing research suggests that TCR signaling in combination with inflammatory cytokines such as IL-27, type 1 interferon (IFN) and IFN-γ, potently drive Ly6C expression (22). Upon further investigation of 2W-specific responses in the SLOs, we found that in contrast to the primary response in which expression of Ly6C and CXCR5 was mutually exclusive, a population of Ly6C^+^CXCR5^+^ Ag-specific cells could be identified throughout the secondary response (Figure 3A). This subset expressed intermediate levels of Ly6C and CXCR5 when compared with CXCR5^–^ Th1 cells and Ly6C^–^ Tfh cells, respectively. Though initially present at a low frequency in the memory Tfh pool, these cells rapidly expanded and could be observed at significantly higher levels in the SLOs and blood of mice during the secondary response (Figures 3B,C). In fact, while the secondary 2W-specific CD4^+^ T cell response in SLOs shifted to a Tfh phenotype (Figures 2H, 3D), this skewing could be reversed by the exclusion of Ly6C^+^ Tfh cells from the analysis (Figure 3D).

Ly6C-expressing Tfh cells have been described in the context of LCMV infection (5). Ly6C is T-bet-regulated, although it can be induced in the absence of T-bet via CD3/CD28 signaling (22–24). It was recently shown using a T-bet reporter mouse strain that a subset of T cells in the germinal center (i.e., Tfh cells) had previously expressed T-bet and that these cells may functionally alter immunoglobulin subclass production (25). To test if T cell expression of T-bet was required for Ly6C expression in the context of secondary Lm infection, we infected mice that specifically lack T-bet expression in T cells (Lck-Cre;T-bet^fl/fl^ mice) with Lm-2W and assessed the CD4^+^ T cell recall responses. As expected, we were unable to detect many Ag-specific T-bet^+^ cells via flow cytometry at 5 dpi in contrast to control T-bet^fl/fl^ mice (Supplementary Figure S3A). In the absence of T-bet^+^ cells, the frequency of CXCR5^+^ Tfh cells was significantly higher than in littermate controls, although the absolute numbers were comparable (Supplementary Figure S3B). In the absence of T-bet, Ly6C expression was no longer detectable on Ag-specific Tfh cells (Figure 3E), and Lck-Cre;T-bet^fl/fl^ mice had significantly fewer Ly6C^+^ 2W + LLO:I-A^b^-specific Tfh cells in the spleen as compared with control T-bet^fl/fl^ mice (Figure 3F).

There is considerable interest in the impact of Tfh polarization on help to B cells. To test if the loss of T-bet and Ly6C^+^ Tfh cells affected the antibody response to Lm-2W, we quantified total anti-Lm and anti-LLO IgG as well as anti-Lm IgM, IgG2b, and IgG2c responses in the sera of Lck-Cre;T-bet^fl/fl^ and control mice following secondary Lm infection. Lck-Cre;T-bet^fl/fl^ mice had similar antibody titers compared with control mice at 10 dpi (Figures 3G,H), suggesting that the loss of T cell-specific T-bet expression did not significantly affect the germinal center response to Lm-2W infection, at least at this early time point. In sum, our data show that T-bet is required for expression of Ly6C on Tfh cells during Lm infection but that this population may not be essential for antibody production during recall responses.

### CD49a^+^ Cells Dominate the Secondary Th1 CD4^+^ T Cell Response and Maintain Residence in the Liver

**FIGURE 4:**
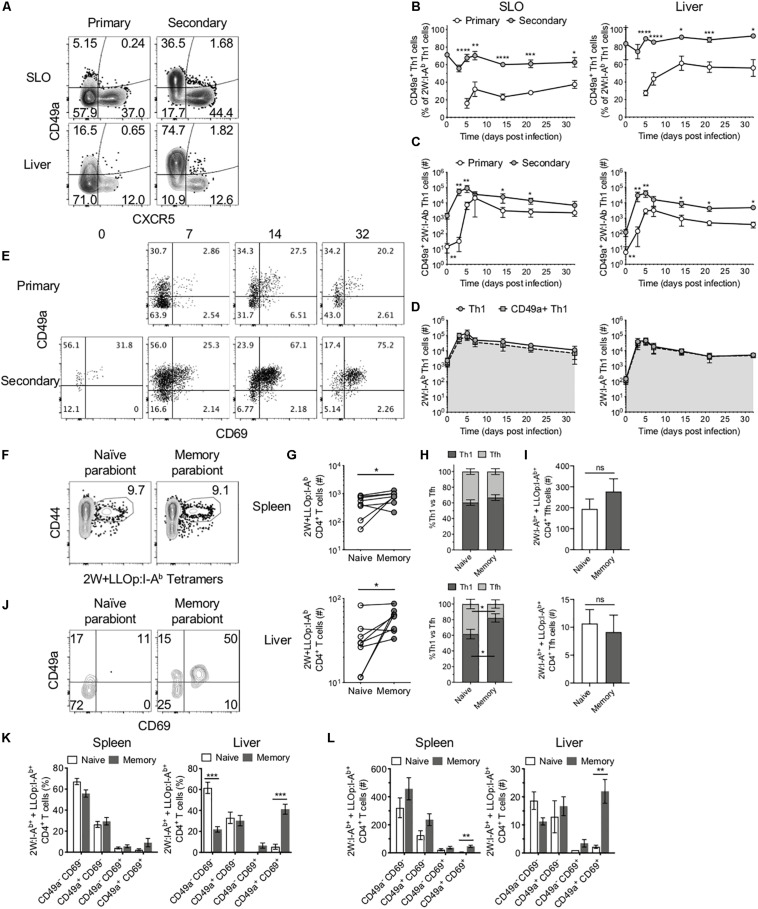
CD49a^+^ cells dominate the secondary Th1 CD4^+^ T cell response and maintain residence in the liver. **(A)** Representative flow cytometry plots demonstrating CXCR5 and CD49a expression on 2W:I-A^b^-specific CD4^+^ cells, excluding Foxp3^+^ Tregs, in the SLOs and liver at 5 dpi following primary or secondary Lm infection. Frequency **(B)** and number **(C)** of CD49a^+^ Th1 cells in the SLOs and liver. **(D)** Number of 2W:I-A^b^-specific Th1 cells (circles) or 2W:I-A^b^-specific CD49a^+^ Th1 cells (squares) in the SLOs and liver following secondary Lm infection. **(E)** Representative flow cytometry plots demonstrating CD69 and CD49a expression on 2W:I-A^b^-specific CD4^+^ Th1 cells in the liver at 0, 7, 14, and 32 dpi following primary or secondary Lm infection. **(F,G)** Lm-immune and naïve mice were conjoined via parabiosis >110 days after Lm infection. Parabiont donors were analyzed 18–19 days after parabiosis. **(F)** Representative flow cytometry plots demonstrating the presence of 2W:I-A^b^ and LLOp:I-A^b^-specific CD4^+^ T cells in the spleens of a parabiont pair. **(G)** Number of 2W:I-A^b^ and LLOp:I-A^b^-specific CD4^+^ T cells in the spleens and livers of parabiont pairs. **(H)** Frequency of 2W:I-A^b^ and LLOp:I-A^b^-specific CD4^+^ T cells exhibiting a Th1 or Tfh phenotype in the spleens and livers of parabiont pairs. **(I)** Numbers of 2W:I-A^b^ and LLOp:I-A^b^-specific CD4^+^ Tfh cells in spleens and livers of parabiont pairs. **(J)** Representative flow cytometry plots demonstrating CD69 and CD49a expression on 2W:I-A^b^ and LLOp:I-A^b^-specific CD4^+^ T cells in the livers of a parabiont pair. Frequency **(K)** and numbers **(L)** of 2W:I-A^b^ and LLOp:I-A^b^-specific CD4^+^ Th1 cells expressing combinations of CD69 and CD49a [as in panel **(J)**] in the spleens and livers of parabiont pairs. Data are compiled from two to three independent experiments per time point with *n* = 1–4 mice per experiment **(A–E)**. Eight parabiont pairs were analyzed **(F–L)**. *P < 0.05, **P < 0.01, ***P < 0.001, ****P < 0.0001 as determined by unpaired Student’s *t*-test **(B)**, Mann Whitney *U* test **(C)**, paired *t*-test **(G)**, two-way ANOVA followed by Sidak’s multiple comparison test **(H,I)**, or matched two-way ANOVA followed by Sidak’s multiple comparison test **(K,L)**. Bars represent mean ± SEM [except panel **(C)**, where bars represent mean ± SD].

In addition to Tfh cells, Th1 cells are the other predominant CD4^+^ T cell subset during Lm infection. Memory Th1 cells rapidly expand upon reinfection, and increased numbers of this subset can be found in the liver following secondary infection (Figure 2B). The liver is a primary site of infection for intravenously delivered Lm, where it may directly infect hepatocytes, placing the host at risk for development of microabscesses in the liver (26). However, the liver also contains specialized macrophages called Kupffer cells that act to clear these bacteria (27). In light of the role that Th1 cells play in supporting macrophage function, we sought to determine whether Lm-specific CD4^+^ T cells exhibit functional differences in secondary responses indicative of enhanced protection in the liver. Indeed, as in SLOs, the numbers of 2W-specific Th1 cells reached a new set point in the liver following secondary infection (Figure 2B). Upon further investigation, we found that while only a small subset of Th1 cells expressed the integrin CD49a at early stages of the primary response, the majority of Th1 cells in the SLOs and liver expressed CD49a during the secondary response (Figures 4A–C). In fact, CD49a expression was gradually acquired by Th1 cells during the primary response to Lm, and this population continued to evolve at memory time points (Figures 4B,C). Numerically, the increase in CD49a^+^ cells accounts almost entirely for the increased number of total 2W-specific Th1 cells in the liver (Figure 4D), suggesting that CD49a is important for CD4^+^ T cell trafficking to the liver or for maintaining residency at this site following Lm infection. In contrast, CXCR5^+^ Tfh cells did not express CD49a in either primary or secondary responses in these tissues (Figure 4A), suggesting that Tfh cells may not form resident populations in the SLOs and liver in the context of Lm infection.

As mentioned, CD49a is often used as a marker of tissue-resident memory T cells (13). CD69 is another marker of residency in tissues such as the liver (8). Kinetic analysis of the expression of CD49a and CD69 on Ag-specific Th1 cells in the liver revealed a population of CD49a^+^CD69^+^ cells that expanded, persisted until memory time points, and could be effectively boosted through reinfection with Lm (Figure 4E). We sought to determine whether these characteristics translated into tissue residency of the CD49a^+^CD69^+^ Th1 subset by performing parabiosis studies to assess whether these cells could transit via the blood to distant sites. In these experiments a naïve mouse (parabiont recipient) was joined to a congenically marked mouse that had been infected with Lm > 110 days previously (parabiont donor) to generate putative liver-resident Th1 cells (Supplementary Figure S2). After 18–19 days, at which time the mouse pairs should have equilibrated their circulatory systems (17), the parabiont pairs were euthanized and the numbers and phenotypes of congenically marked pooled 2W:I-A^b^- and LLOp:I-A^b^-specific (2W + LLOp:I-A^b^) CD4^+^ T cells were assessed in each mouse. 2W and LLOp tetramer-binding cells were detected in the spleens of both mice in each pair (Figure 4F). The numbers of Ag-specific CD4^+^ T cells were higher in the spleens and livers of the memory (parabiont donor) mice as compared with the naïve (parabiont recipient) mice, consistent with tissue-residency of one or more Ag-specific CD4^+^ T cell subsets (Figure 4G). Th1 and Tfh subsets were present at similar frequencies in the spleens of parabiont donor and recipient mice (Figures 4H,I), suggesting that in the spleen these populations were comprised of largely non-resident cells. In contrast, the livers of memory parabionts showed an enrichment of Th1 cells and the livers of naïve parabionts exhibited a concomitant increase in Tfh cell frequency (Figure 4H), in agreement with a large liver-resident Th1 subset that fails to effectively recirculate through the bloodstream. Consistent with this possibility, and with the observed phenotypes in intact mice (Figure 4E), congenically marked CD49a^+^CD69^+^ Th1 cells were found almost exclusively in the livers of memory mice as compared with naïve parabionts (Figures 4J–L), suggesting that the CD49a^+^CD69^+^ Th1 cells were resident in the liver and did not recirculate efficiently. While CD49a^+^CD69^+^ Th1 cells were also significantly enriched in the spleens of memory parabiont mice consistent with tissue residency (Figure 4L), this subset comprised only a minority of splenic Th1 cells, in agreement with the observation that most splenic Th1 cells appeared to recirculate through the bloodstream (Figure 4H).

Together, these data demonstrate that in contrast to Ag-specific Tfh cells and splenic Th1 cells that efficiently recirculate, Th1 cells in the liver include a large resident CD49a^+^CD69^+^ subset that emerges during the primary response, can be specifically boosted during the secondary response, and persists to achieve a new setpoint within this tissue.

## Discussion

We assessed the intact Ag-specific CD4^+^ T cell response after primary and secondary bacterial infection. Our results demonstrate that upon recall, Ag-specific CD4^+^ T cells expand and then contract more quickly than during a primary infection. These data align with other studies that have shown that memory CD4^+^ T cells have reduced proliferative potential compared with naïve T cells (28). Similarly, secondary memory CD8^+^ T cells expand less than primary memory CD8^+^ T cells during Lm or LCMV-Armstrong infection (29), although precursor frequency affects memory CD8^+^ T cell proliferative potential following boosting (30). Primary Lm infection generates two main classes of effectors: Th1 cells that assist macrophages and Tfh cells that aid B cells in antibody production. We found that after secondary infection, Tfh cells expanded to a modestly greater degree than Th1 cells, and that phenotypic differences emerged in both cell types.

Several studies have provided evidence supporting the existence of memory Tfh cells (5, 20, 21, 31). Our work revealed that during Lm infection, a small subset of Ag-specific Tfh cells expressed the marker Ly6C during the primary infection and maintained expression at memory time points; however, upon reinfection, this population rapidly expanded in SLOs. Ly6C is a GPI-anchored membrane glycoprotein that can act as a costimulatory molecule for T cells (32). Hale and colleagues found that, although less prominent during the primary infection, CXCR5^+^Ly6C^int^ cells expanded more extensively than CXCR5^+^Ly6C^–^ cells following transfer and re-challenge with LMCV (5). However, both subsets gave rise to Tfh effector cells, suggesting that both subsets are memory cells. We found that following secondary Lm infection, more CXCR5^+^ Tfh cells expressed Ly6C, and the Ly6C^+^ Tfh population was the basis for the Tfh-skewing observed in SLOs during the secondary response. Additionally, genetic ablation of T-bet resulted in loss of Ly6C expression, indicating that Ly6C is T-bet-regulated in this setting.

Following adoptive transfer, secondary memory CD8^+^ T cells have been shown to be better at clearing acute bacterial (Lm) and viral (LCMV, VacV) infections in mice but are less functional than primary memory CD8^+^ T cells against chronic LCMV infection (29, 33). CD4^+^ T cells play important roles in controlling bacterial and viral infections through the production of cytokines (predominantly secretion of IFN-γ by Th1 cells) and by stimulating B cells to produce antibodies (Tfh cells). Given the increased frequency and number of Ly6C^+^ Tfh cells following secondary Lm infection, we assessed antibody responses to Lm in the presence or absence of this subset. We found that loss of the Ly6C^+^ Tfh cells, due to genetic deletion of T-bet, did not affect the antibody response to Lm-2W. These data suggest that either Ly6C expression does not mark Tfh cells with special antibody-promoting capacity or other Tfh cell subsets compensated for the loss of Ly6C^+^ Tfh cells. A recent report identified Th1-derived Tfh-like cells that co-expressed T-bet and Bcl6 and stimulated antibody production from B cells to similar levels as conventional Tfh cells (34). This suggests that multiple Tfh phenotypes exist that can promote B cell responses. Further characterization of Tfh memory cells, perhaps using single-cell approaches, is needed to determine whether the variability of Tfh memory is due to cellular plasticity or population heterogeneity, or both. For instance, a recent report used single-cell RNA-sequencing to identify folate receptor 4 (FR4) as a marker of long-lived Tfh cells following LCMV infection (20). Precursors of CXCR5^+^ Tfh populations show greater potential to develop into memory cells compared with Th1 precursors and share gene-expression signatures with memory CD8^+^ T cells, suggesting that CXCR5^+^ Tfh-derived memory cells have greater plasticity in generating secondary effector phenotypes (4, 20, 35). Recently, Cuicci et al. used single-cell RNA-sequencing to evaluate LCMV-specific CD4^+^ T cells heterogeneity during acute infection (36). Their data support the idea that memory Tfh multi-potency may be a result of population heterogeneity.

In light of the emerging importance of tissue-resident memory T cells in conferring protection against certain infections and our observation that the number of Th1 cells in the liver reached a new set point following secondary Lm infection, we assessed the phenotype of these cells. Multiple studies have shown that murine and human CD4^+^ and CD8^+^ tissue-resident memory T cells express the integrin CD49a, most often in the lung and skin (9, 11, 13, 37). CD49a also marks a subset of liver-resident NK cells and macrophages with unique properties and may play a role in regulating liver inflammation (10, 12, 14). CD49a (integrin α1) binds to CD29 (integrin β1) from VLA-1, an integrin specific to collagen (38), suggesting that CD49a may be important for adhesion of cells to the collagen-rich basement membrane of epithelial cells. Indeed, antibody blockade or genetic deletion of VLA-1 in mice results in reduced retention of tissue-resident memory CD8^+^ T cells in peripheral tissues (39). A similar phenotype (CD69^+^CD49a^+^) was found for tissue-resident memory CD4^+^ T cells in non-lymphoid tissues following LCMV infection (40). We found that while only a small fraction of T-bet^+^ Th1 cells in the liver co-expressed the markers CD49a and CD69 during early stages of primary Lm infection, this population was enriched over time, could be rapidly boosted through reinfection with Lm, and persisted at higher numbers and frequency within the liver post-secondary infection. To assess the residency of Th1 cells in the liver during Lm infection, we performed parabiosis studies, which indicated that in contrast to Tfh cells and most splenic Th1 cells, a significant fraction of Ag-specific Th1 cells in the liver co-expressed CD49a and CD69 and maintained residence in this tissue. Tissue-resident memory T cells have also been reported to express CD103 and CXCR6 in non-lymphoid tissues (8, 41). We did not examine expression of these markers by the Ag-specific CD49a^+^CD69^+^ Th1 subset in the liver in this study. However, liver-resident memory CD4^+^ T cells were not found to express CD103 following LCMV infection (40). CXCR6 is part of a core cluster of genes that define CD69^+^ resident memory CD8^+^ T cells in mice and humans (11, 37, 42) and is required for the maintenance of liver-resident CD8^+^ T cells following immunization with *Plasmodium* sporozoites or vaccinia virus (43). Assessment of these markers in conjunction with immunohistochemical analyses could further strengthen the conclusions of this study. Tissue-resident memory T cells provide immunosurveillance and localized protective immunity in non-lymphoid tissues, and our data corroborate other studies that have demonstrated that prime-boost strategies can increase tissue-resident memory T cell numbers and function in these sites (8, 44). A better understanding of the mechanisms mediating the differentiation of CD4^+^ tissue-resident memory T cells will allow us to harness the protective capacity of this memory population and modulate their activity in the context of infection or inflammatory diseases.

In summary, we assessed the response of CD4^+^ T cells following primary and secondary Lm infection in the same host. The kinetics of the responses were largely similar, although the secondary response peaked and contracted more quickly than the primary response. Importantly, we identified phenotypic differences in both Th1 and Tfh cells following secondary infection. The majority of our successful vaccines rely on neutralizing antibody and long-lived humoral responses for protective immunity (45). Thus, gaining a better understanding of the development, function, and contribution of Tfh cells within the context of prime and boost vaccination and pathogen challenge will provide avenues for rational vaccine design. Moreover, tissue-resident memory T cells provide protective immunity in non-lymphoid tissues, and various strategies have been developed to specifically target these cells to tissues (46). Most work has focused on tissue-resident memory CD8^+^ T cells, although a recent study demonstrated the existence and function of tissue-resident memory CD4^+^ T cells in mice (40). Thus, our data will help inform the design of strategies that promote CD4^+^ tissue-resident memory T cells and/or Tfh cells with specific phenotypes or functions.

## Data Availability Statement

The raw data supporting the conclusions of this article will be made available by the authors, without undue reservation.

## Ethics Statement

The animal study was reviewed and approved by University of Minnesota Institutional Animal Care and Use Committee.

## Author Contributions

DM conceived and designed the study, performed experiments, analyzed the data, obtained funding, generated figures, and critically revised the manuscript. KB conceived and designed the study, performed experiments, analyzed the data, generated figures, and wrote the manuscript. MJ and AF conceived and designed the study, obtained funding, and critically revised the manuscript. All authors read and approved the final manuscript.

## Conflict of Interest

The authors declare that the research was conducted in the absence of any commercial or financial relationships that could be construed as a potential conflict of interest.
