# Supplementary material for: Antigen-Specific CD4+ T Cells Exhibit Distinct Kinetic and Phenotypic Patterns During Primary and Secondary Responses to Infection
Source: Front Immunol. 2020 Sep 2;11:2125. doi: 10.3389/fimmu.2020.02125 (PMC7492679; doi:10.3389/fimmu.2020.02125)
Supplement: Supplementary file 1 [file Data_Sheet_1.PDF]

Contribution to the Field Statement

After encountering an infection for the first time, the adaptive immune system generates memory cells that may respond more quickly and robustly upon re-exposure, protecting the host by preventing or shortening the duration of secondary infection. Understanding how memory cells respond to re-exposure to a pathogen is critical for designing efficient vaccination strategies. While prior work has explored the fates of antigen-specific $CD4^+$ T cell subsets at acute and memory timepoints, there has been minimal investigation of intact memory responses relative to the primary response, limiting our understanding of specific changes in the kinetics and quality of memory responses. We quantified and characterized unmanipulated, antigen-specific $CD4^+$ T cell responses throughout primary and secondary infection with the bacterial pathogen *Listeria monocytogenese* (Lm). Antigen-specific $CD4^+$ T cells expanded more quickly in secondary lymphoid organs and the liver during secondary responses, reaching a new set point in the liver, a site of intravenous Lm infection. Additionally, we identified two new subsets of $CD4^+$ T cells that preferentially expanded during secondary infection; one maintained residence in the liver and the other in secondary lymphoid organs. Our data demonstrate the accelerated kinetics and altered differentiation of $CD4^+$ T cells associated with secondary responses to infection.
