# Supplementary material for: Antigen-Specific CD4+ T Cells Exhibit Distinct Kinetic and Phenotypic Patterns During Primary and Secondary Responses to Infection
Source: Front Immunol. 2020 Sep 2;11:2125. doi: 10.3389/fimmu.2020.02125 (PMC7492679; doi:10.3389/fimmu.2020.02125)
Supplement: Supplementary file 2 [file Data_Sheet_2.PDF]

Supplementary Material

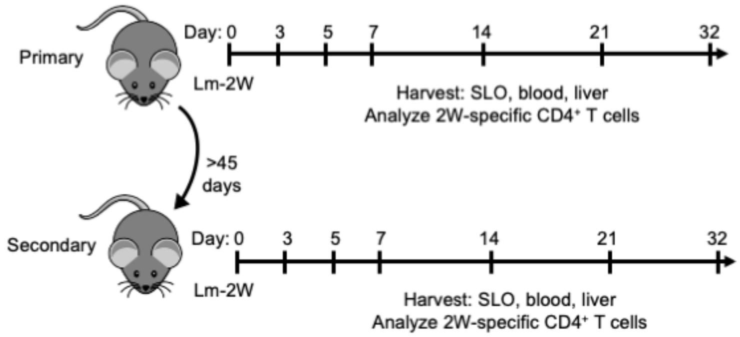

Supplementary Figure 1. Analysis of Ag-specific CD4+ T cells following primary and secondary Lm infection. Schematic describing the experimental set up to compare antigen-specific CD4+ T cell responses following primary or secondary Lm infection of intact mice.

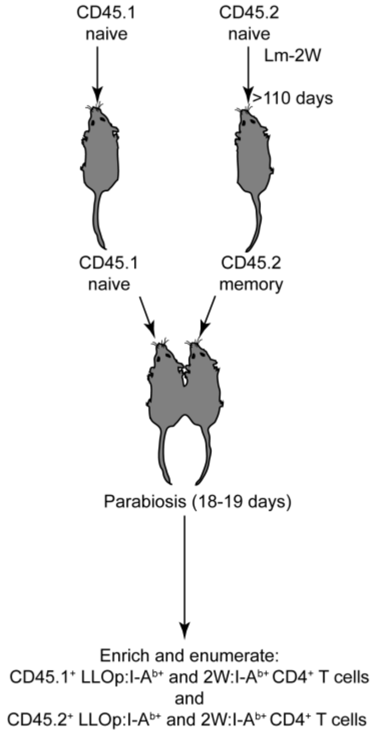

Supplementary Figure 2. Analysis of Ag-specific CD4+ T cells following parabiosis. CD45.2+ mice were infected with Lm-2W; then, >110 dpi, mice were conjoined to CD45.1+ naïve mice via

parabiosis. 18-19 days after surgery, 2W:I-A^b and LLO:I-A^b-specific $CD4^+$ T cells were enriched from SLO and liver of each parabiont and analyzed via flow cytometry.

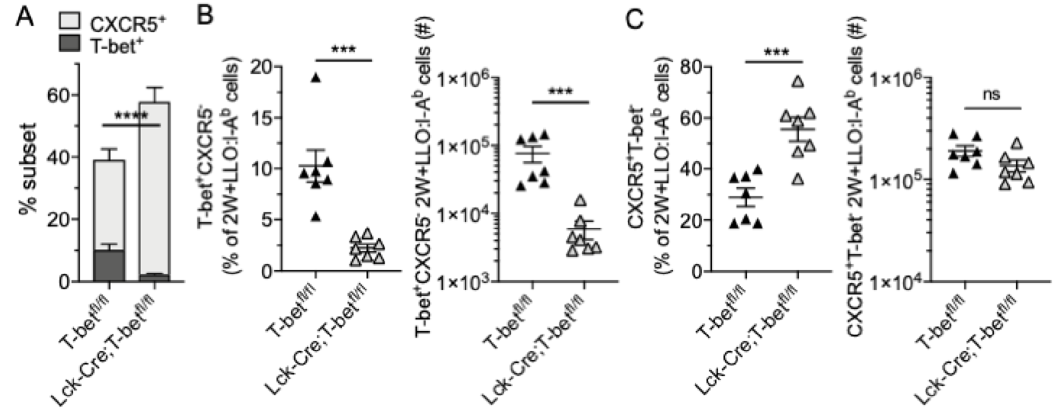

Supplementary Figure 3. Lck-Cre;T-bet$^{fl/fl}$ mice have reduced Ag-specific T-bet$^+$ Th1 cells following secondary Lm infection. (A) Following secondary Lm infection, the frequency of 2W+LLO:I-A^b-specific T-bet$^+$ Th1 cells or CXCR5$^+$ Tfh cells was assessed in SLO of T-bet$^{fl/fl}$ or Lck-Cre;T-bet$^{fl/fl}$ mice at 5 dpi. Number of 2W:I-A^b–specific Th1 (B) or Tfh (C) cells in SLO following secondary infection of T-bet$^{fl/fl}$ or Lck-Cre;T-bet$^{fl/fl}$ mice at 5 dpi.
